# Supplementary material for: Oxygen Consumption and Basal Metabolic Rate as Markers of Susceptibility to Malignant Hyperthermia and Heat Stroke
Source: Cells. 2022 Aug 9;11(16):2468. doi: 10.3390/cells11162468 (PMC9406760; doi:10.3390/cells11162468)
Supplement: Supplementary file 1 [file cells-11-02468-s001.zip › cells-1826012-supplementary.pdf]

Genotype	N° of animals	Core temperature (°C)	Weight (g)	Food intake (g/day)
C57bl6	10	35.57 ± 0.30	24.74 ± 0.57	3.27 ± 0.05
CASQ1-null	8	36.26 ± 0.09	23.80 ± 0.58	3.88 ± 0.02 **
Y522S	8	36.14 ± 0.16	23.09 ± 0.55	4.05 ± 0.09 **

Table S1. Core temperature, body weight and food intake. Core temperature, body weight, and food intake of 2 months old mice. Data are shown as mean ± SEM (**$p < 0.01$ in comparison with C57bl6), as evaluated by one-way ANOVA followed by Tukey's post-hoc test.
